# Comparison of the within-reader and inter-vendor agreement of left ventricular circumferential strains and volume indices derived from cardiovascular magnetic resonance imaging

**DOI:** 10.1371/journal.pone.0242908

**Published:** 2020-12-15

**Authors:** Doyin S. Mansell, Evelyn G. Frank, Nathaniel S. Kelly, Bruno Agostinho-Hernandez, James Fletcher, Vito D. Bruno, Eva Sammut, Amedeo Chiribiri, Thomas Johnson, Raimondo Ascione, Jonathan W. Bartlett, Harinderjit S. Gill, Katharine H. Fraser, Andrew N. Cookson

**Affiliations:** 1 Department of Mechanical Engineering, University of Bath, Bath, United Kingdom; 2 Department for Health, University of Bath, Bath, United Kingdom; 3 Bristol Heart Institute and Translational Biomedical Research Centre, Faculty of Health Science, University of Bristol, Bristol, United Kingdom; 4 School of Biomedical Engineering and Imaging Sciences, King's College London, London, United Kingdom; 5 Department of Mathematical Sciences, University of Bath, Bath, United Kingdom; Karolinska Institutet, SWEDEN

## Abstract

**Purpose:**

Volume indices and left ventricular ejection fraction (LVEF) are routinely used to assess cardiac function. Ventricular strain values may provide additional diagnostic information, but their reproducibility is unclear. This study therefore compares the repeatability and reproducibility of volumes, volume fraction, and regional ventricular strains, derived from cardiovascular magnetic resonance (CMR) imaging, across three software packages and between readers.

**Methods:**

Seven readers analysed 16 short-axis CMR stacks of a porcine heart. Endocardial contours were manually drawn using OsiriX and Simpleware ScanIP and repeated in both softwares. The images were also contoured automatically in Circle CVI42. Endocardial global, apical, mid-ventricular, and basal circumferential strains, as well as end-diastolic and end-systolic volume and LVEF were compared.

**Results:**

Bland-Altman analysis found systematic biases in contour length between software packages. Compared to OsiriX, contour lengths were shorter in both ScanIP (-1.9 cm) and CVI42 (-0.6 cm), causing statistically significant differences in end-diastolic and end-systolic volumes, and apical circumferential strain (all p<0.006). No differences were found for mid-ventricular, basal or global strains, or left ventricular ejection fraction (all p<0.007). All CVI42 results lay within the ranges of the OsiriX results. Intra-software differences were found to be lower than inter-software differences.

**Conclusion:**

OsiriX and CVI42 gave consistent results for all strain and volume metrics, with no statistical differences found between OsiriX and ScanIP for mid-ventricular, global or basal strains, or left ventricular ejection fraction. However, volumes were influenced by the choice of contouring software, suggesting care should be taken when comparing volumes across different software.

## Introduction

Accurate and reproducible quantification of cardiovascular properties is imperative for correct identification and management of many cardiovascular diseases. Prognosis post myocardial infarction is partially based on measurements of end-diastolic and end-systolic volumes, or left ventricular ejection fraction (LVEF), as these reflect adverse remodelling of the left ventricle [1–4]. Non-invasive measurement techniques using many imaging modalities have become popular in monitoring disease progression. Cardiovascular magnetic resonance (CMR), for example, provides high spatial resolution, has a high reproducibility, especially for images taken in the short-axis [5, 6], and is now widely used for quantification of clinical measurements by both clinical and research institutions.

CMR images can be used as non-invasive diagnostic tools to assess LVEF and, more recently, ventricular strains. Ventricular strains quantify ventricular deformation and are dependent on the change in endocardial or epicardial lengths throughout the cardiac cycle. They have been shown to be sensitive markers for myocardial dysfunction [5, 7, 8]. Strain can be measured from imaging data such as CMR, usually using off-line post-processing software packages. Most strain methods, like LVEF, depend on the placement of endocardial or epicardial contours, thus accurate and repeatable contouring is needed for precise results. There are three factors affecting the precision of strain acquisition: the repeatability of imaging/image quality, the choice of software used for image processing and contouring, and the reader confirming or altering the resultant contours [9, 10]. Poor image quality, especially in short-axis images, can be minimised by use of CMR imaging as opposed to echocardiography [6]. Choice of software and individual reader bias have been shown to produce differences in contouring, which in turn lead to differences in left ventricular volumes [9, 11]. Inconsistencies in strains, nevertheless, have not been robustly investigated; as strains are derived from the same endocardial/epicardial border data used for the calculation of LVEF, it is possible that there will also be inconsistencies in strain values too.

A large range of commercial software packages have become available over the last decade, many with in-built image analysis tools, which can operate on an automatic, semi-automatic, or manual basis. Through machine learning and greyscale thresholding techniques, vendors have been able to incorporate automatic and semi-automatic contouring of the left ventricle into software packages, with accurate results which need minimal manual alterations [12]. Calculation of strain should be simple if contour lengths are known and can be done relatively simply with numerical analysis software, however many advanced software packages have in-built strain calculation. This calculation may rely on placement of landmarks such as right ventricular insertion points to the left ventricle, increasing the need for human input. Moreover, these are typically ‘black box’ systems using closed-source, proprietary algorithms for image segmentation and/or strain calculation, which increases the uncertainty of the strain calculation.

A cross-centre study for CMR centres showed that there can be large variations in both endocardial and epicardial contouring even amongst expert readers [11], confirming that delineation of the left ventricular myocardium from the blood pool and epicardium is highly dependent on the reader, even when using images of superior quality such as steady-state free procession CMR images [9]. This potentially results in differences in final clinical metrics. It has even been noted that until now, there has been inconsistency and poor repeatability in strain calculations with few validated values for circumferential strain especially, and no universal absolute values for healthy or different pathological strains [13–15], which have limited translation into clinical decision making and standard practice guidelines [16].

This study aimed to investigate the variability introduced to clinical metrics by different readers and different pieces of software when using CMR images. The clinical metrics under investigation were end-diastolic volume (EDV), end-systolic volume (ESV), LVEF, global circumferential strain (GCS), and three regional circumferential strains; apical, mid-ventricular, and basal.

## Methods

### Data acquisition

Cine CMR images from one porcine specimen (age = 7 months) suffering from induced heart failure with dilated cardiomyopathy were selected. This data collection, and analysis presented here, was part of a study demonstrating a closed-chest porcine model of myocardial infarction and reperfusion, assessed by serial imaging and biomarker measurements, and proteomics tissue analysis [17]. Those animal procedures were undertaken at the University of Bristol Translational Biomedical Research Centre in accordance with the United Kingdom Animal (Scientific Procedures) Act, 1986 (under PPL 7008975) granted by the Home Office after formal review and approval by the University of Bristol Animal Welfare and Ethics Review Body (AWERB). Specimens were induced to general anaesthesia by a professional veterinary anaesthetist, using an IV infusion of Propofol at 1.0 mg/kg boluses, with anaesthesia maintained with Isoflurane in oxygen with the vaporiser set at 2%. Finally, while still under anaesthesia and full monitoring, animals underwent median sternotomy to expose the heart with euthanasia involving intracoronary injection of cold (0-4°C) cardioplegic solution.

CMR images were acquired in both short- and long-axes slices, using a CMR image scanner (Siemen Healthcare Limited, Magnetom 3T PRISMA, Erlangen Germany) with a spatial resolution of pixels of 1.6 mm x 1.6 mm. Temporal resolution was 30 frames per cycle. FISP functional cinematic images were acquired in the three long axis planes (2-,3- and 4- chamber orientation) of the left ventricle and in a contiguous stack of short axis slices from base (at the level of the mitral valve) to the apex. The acquisition parameters are as follows: slice thickness 8mm, base resolution 208, phase resolution 80%, TE 1.39 ms, TR 46.5 ms, flip angle 45o, bandwidth 1335 Hz/Pixel, calculated phases (temporal resolution) 30, data segments per R-R 15. For short-axis images the slice thickness was 8 mm with a 2 mm gap, which is similar to that used in other studies [17–19]. The short-axis view series covered the left ventricle from the apex to the base using 11 slices, nine of which were chosen for contouring in this study. Data were taken from one specimen, at one time point from a larger study [20], and the data were from the 30-day experimental time point, as the image quality was considered to be superior with no image artefacts.

### Image analysis

**Fig 1:**
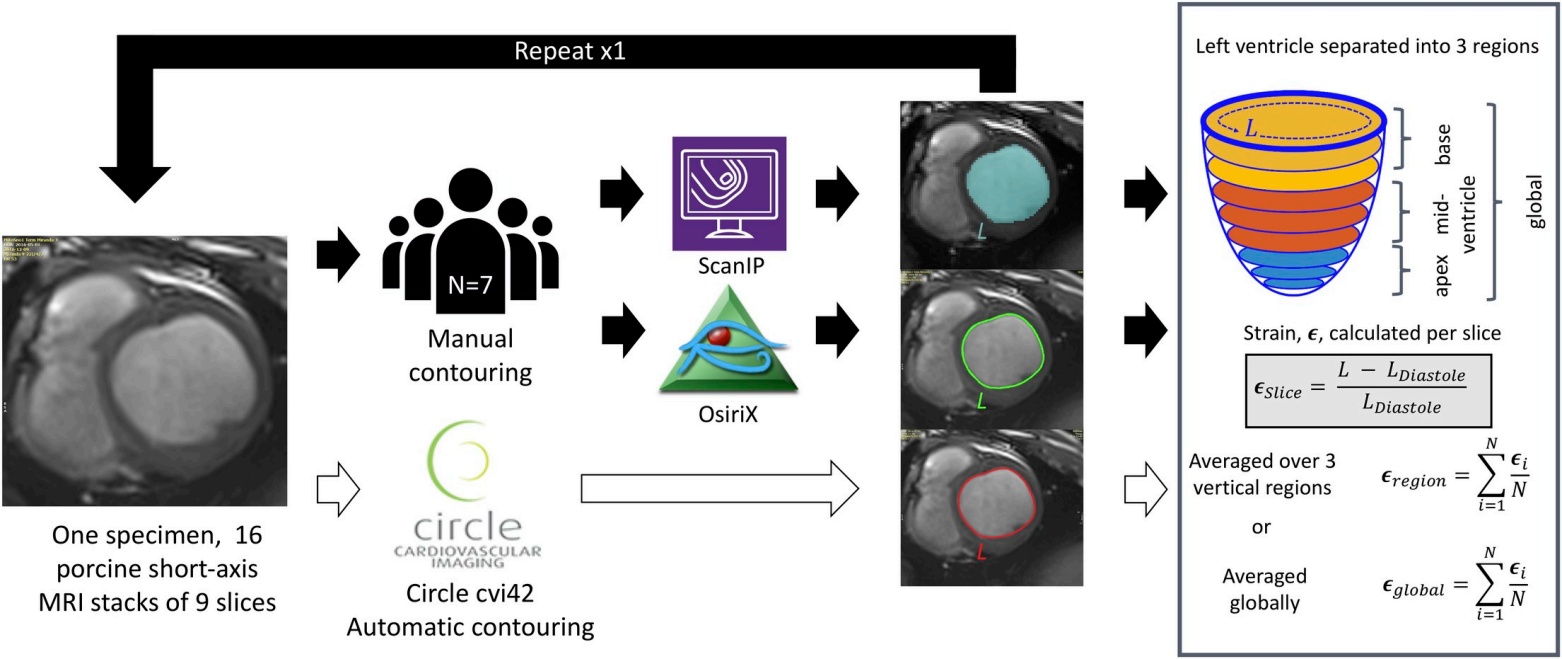
Schematic representation of workflow from imaging through to strain analysis. 16 short-axis cardiac MRI stacks were used from one specimen. Endocardial contours were traced in three software packages ScanIP, OsiriX, and Circle CVI42. Short-axis slices were grouped into three regions apex, mid-ventricle, and base, and regional circumferential strains were calculated as the average over slices in the region. Global circumferential strain, end-diastolic and end-systolic volumes, and left ventricular ejection fraction were also calculated.

Seven readers from one institution (University of Bath, Bath, UK) independently analysed the data set on four separate occasions, twice on each of two software packages (OsiriX and ScanIP) with at least one interval passing between each occasion. The whole workflow is shown as a schematic in Fig 1.

**Fig 2:**
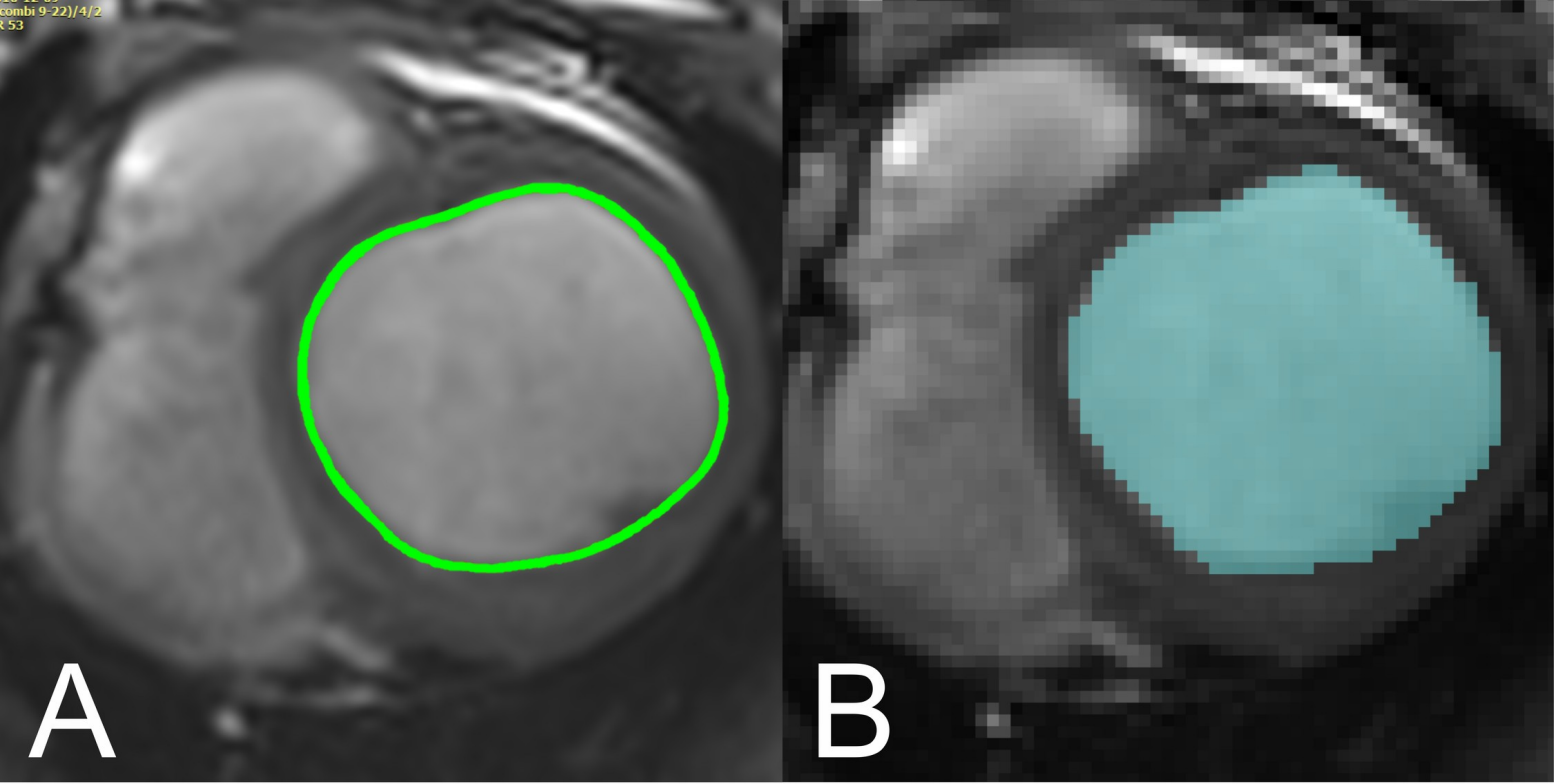
Visual comparison of contouring approaches in ScanIP and Osirix. A. Contour representation in ScanIP. Pixels within the endocardium are selected (based on thresholding or manual selection), to be exported in a binary image format. B. Contour representation in OsiriX. Contours markers are placed at points around the endocardium, and a spline curve is interpolated between these points.

OsiriX (Pixmeo, Geneva, Switzerland) is a DICOM viewer commonly used in clinical settings. The user places a number of markers along the border of interest with the software then interpolating a spline curve between these points to generate the contour (Fig 2A). The length and internal area of this contour are given automatically in cm and cm^2^ respectively. In OsiriX, typically one cine CMR image slice is viewed through all frames, showing the user the same location at different points in the cardiac cycle as references to guide their contouring.

Simpleware ScanIP (Synopsys, Inc., Mountain View California, USA, Version 7) is a 3D image segmentation and processing software commonly used in biomedical engineering, and which has a comprehensive environment capable of handling and complying with DICOM standards. Segmentation/contouring is based on thresholding the greyscale values of the DICOM images, and individual pixels can be inserted or removed using a ‘paint’ tool (Fig 2B). There is no inbuilt tool to measure curved lengths and perimeters, so images were binarised and exported for analysis in MATLAB (Release 2017b, The MathWorks, Inc., Natick, Massachusetts, United States). The convex hull of this segmented region of interest was found and the total perimeter length of the convex hull calculated. In ScanIP all slices at one point in the cardiac cycle can be viewed in order to provide a 3D representation, but neighbouring slices are presented as references to guide the user’s contouring.

Most readers had prior knowledge of at least one of the pieces of software or were otherwise trained on the software prior to the study taking place. Two users had prior knowledge of delineating LV contours and a training session on this process was provided to the other users prior to testing. However, four of the users did have prior knowledge of the ScanIP software (one for LV contouring) and one user prior knowledge with OsiriX again for LV contouring. The experienced reader was equally experienced in both software packages. All contours were placed manually in both OsiriX and ScanIP, following the SCMR guidelines of standardised image interpretations and post-processing of DICOM CMR images [21], with both papillary muscles and trabeculae included in the blood pool. All readers received training in the software new to them and in the SCMR contouring guidelines from one expert reader before undertaking the study; however, all readers performed their analyses in isolation, with no further influence.

Sixteen consecutive short axis CMR frames from the original 30 were chosen for endocardial contouring, with the first frame occurring just before end-diastole (ED) and the last frame just after end-systole (ES). Readers contoured all 9 slices from the 16 timeframes provided to them, covering the majority of the LV, from base to apex. All contours were assessed by an experienced reader to ensure they followed the SCMR guidelines of the experiment and were otherwise clinically acceptable.

Once all contours were drawn, the data were extracted from OsiriX and ScanIP and processed using an automated MATLAB script. Volumes for each frame were calculated for both OsiriX and ScanIP. OsiriX automatically calculates the area enclosed by a contour for each slice and frame. These areas were multiplied by the sum of the image slice thickness (8 mm) and gap (|2 mm), i.e. 10 mm, and summed across the LV to give the total LV volume. This is the composite midpoint integration method described by O’Dell et al. [22] and was found in that study to be sufficiently accurate for clinical use when used with between six to twelve image slices.

For ScanIP the MATLAB function *convhull* returned the area within the convex hull derived for each contour. These areas were similarly summed for each slice and frame, and then multiplied by 10 mm to find the volumes at each time-point. For the calculation of clinical metrics, ED was defined as the time with maximum LV volume, and ES was defined as the time with minimum LV volume. From these volumes, LVEF was calculated.

Endocardial circumferential strain was also calculated using the perimeter length data for every slice and frame for both OsiriX and ScanIP. Strain, *ϵ_n_*, was defined using the clinical standard, in Eq 1:

$$\epsilon_{n}=\frac{L_{n}-L_{0}}{L_{0}}$$ (1)

where *L*_0_ is the endocardial perimeter reference length at ED, and *L_n_* is the endocardial perimeter length at time *n* for a given cine slice. These short axis image slices were then grouped into three vertical regions in the LV, apex, mid-ventricle, and base, with each region containing three slices. The means of the circumferential strains in each region were then calculated to give: apical circumferential strain (ACS), mid-ventricular circumferential strain (MCS), basal circumferential strain (BCS) [6]. GCS was also calculated as the mean of the circumferential strains from all nine slices.

CVI42 (Release 5.11.2, Circle Cardiovascular Imaging Inc., Calgary, Canada), which has an in-built automatic contouring function, was also tested on the same CMR images. Contouring was performed once using the software’s automated algorithm and these contours were then used to calculate endocardial strain as before. CVI42 has the functionality to calculate clinical indices such as LVEF from these contours, and although it can also calculate myocardial strain, the strain definition it uses is quite different to that defined here and thus would not form a suitable comparison. Using CVI42’s automatic contouring of the endocardium in this way does however still provide an ‘automated method’ for computing the strain metrics of ACS, MCS, BCS, and GCS for comparison with that derived from OsiriX and ScanIP. Perimeter lengths, areas, volumes, and strains were determined as before, with all measurements and post-processing undertaken by one experienced reader. The results from this CVI42-based method were then directly compared to those from OsiriX and ScanIP.

### Statistical analysis

Data are presented as means, standard deviations, and 95% confidence intervals for each software package and repetition. Mean differences and standard deviations between the four groups are also presented.

Statistical analyses were run on all variables of interest: ACS, MCS, BCS, GCS, EDV, ESV, and LVEF. Paired t-tests were used to compare means, either between ScanIP and OsiriX or between reader contouring repetitions. T-tests pairing OsiriX repetition one with OsiriX repetition two, and pairing ScanIP repetition one with ScanIP repetition two were performed to assess within-software variability. T-tests pairing OsiriX repetition one with ScanIP repetition one, and OsiriX repetition two with ScanIP repetition two were performed to assess inter-software package differences. A p-value of 0.05 was the desired level of statistical significance, however, as several parameters were being assessed, Bonferroni corrections were performed to decrease the likelihood of type I errors [17], yielding a significant p-value ≤ 0.007. As CVI42 gave only one data point for each clinical index it could not be included in these statistical analyses, and therefore only the contour length data was compared using Bland-Altman analysis.

Bland-Altman analysis was performed to evaluate the variation and bias in the endocardial perimeter length measurements across all the readers, with limits of agreement defined at 95%. The Bland-Altman analysis data points were split into test repetitions when comparing ScanIP to OsiriX (test 1 and test 2). As CVI42 had only one test repetition, the assumption was made that CVI42 could be considered 100% repeatable in the delineation of the endocardial contours, as they were generated automatically. As such, the CVI42 data was treated as both the first and second test repetitions for comparison with the first and second test repetitions in ScanIP and OsiriX. All statistical analyses were performed using Stata (StataCorp. 2019. Stata Statistical Software: Release 16. College Station, TX: StataCorp LLC.) and MATLAB.

## Results

### Visual assessment of contouring

After visual assessment of all contours from all readers by one expert reader, no unacceptable contours were found in ScanIP or OsiriX. It was also noted qualitatively that the locations of large disagreement between readers were most noticeable at the apex. CVI42 performed well for the porcine data, although it is not currently validated for use in animal models. Manual alteration of these automatically derived contours was performed when required, mainly in the apex. It was also found that CVI42 had not accurately identified the time points corresponding to ES and ED from the images, so these were judged by one expert reader.

### Mean and standard deviation of attempts

Means of each variable, for each group, (OsiriX test 1 and 2, and ScanIP test 1 and 2) and their standard deviations are presented in Table 1. Comparison of the means for a particular variable across the four groups provides a comparison between each software and each attempt. Similar means are found for across the four groups for MCS, BCS, GCS and LVEF, however slightly larger variations are observed for ACS, and much larger variation for EDV and ESV. This analysis is made more precise in the next section and Table 2.

**Table 1:** Means, standard deviations, standard deviation as a percentage of the mean, and 95% confidence intervals for all variables in each software package for both repetitions.

Variable	Test	Mean	SD	SD as a % of mean	Lower 95% CI	Upper 95% CI
**ACS (%)**	OsT1	-11.9	4.1	34.5	-15.7	-8.2
	OsT2	-10.5	2.2	21.0	-12.6	-8.5
	ScT1	-15.9	3.1	19.5	-18.8	-13.1
	ScT2	-13.5	2.2	16.3	-15.4	-11.4
**MCS (%)**	OsT1	-19.7	1.1	5.6	-20.8	-18.7
	OsT2	-18.8	1.3	6.9	-20.0	-17.6
	ScT1	-21.1	1.7	8.1	-22.6	-19.5
	ScT2	-20.6	1.0	4.9	-21.6	-19.7
**BCS (%)**	OsT1	-30.1	0.9	3.0	-30.9	-29.3
	OsT2	-29.5	1.52	5.1	-30.9	-28.1
	ScT1	-30.5	2.6	8.5	-32.9	-28.0
	ScT2	-29.8	3.1	10.4	-32.7	-27.0
**GCS (%)**	OsT1	-20.0	1.4	7	-21.3	-18.8
	OsT2	-19.7	1.0	5.1	-20.6	-18.7
	ScT1	-20.9	1.8	8.6	-22.6	-19.2
	ScT2	-20.3	1.8	8.9	-22.0	-18.7
**EDV (mL)**	OsT1	160.1	8.0	5.0	152.7	167.4
	OsT2	158.7	9.9	6.2	149.5	167.8
	ScT1	123.8	7.8	6.3	116.6	131.0
	ScT2	122.8	7.5	6.1	115.9	129.8
**ESV (mL)**	OsT1	98.1	8.1	8.3	90.6	105.6
	OsT2	97.6	9.5	9.7	88.9	106.4
	ScT1	74.8	7.9	10.6	67.5	82.1
	ScT2	76.0	6.9	9.1	69.6	82.4
**LVEF (%)**	OsT1	38.9	2.3	5.9	36.8	41.0
	OsT2	38.6	2.6	6.7	36.2	41.0
	ScT1	39.6	3.3	8.3	36.5	42.6
	ScT2	38.1	2.5	6.6	35.9	40.4

OsT1 = OsiriX test 1, OST2 = OsiriX test 2, ScT1 = ScanIP test 1, ScT2 = ScanIP test 2, ACS = apical circumferential strain, MCS = mid-ventricular circumferential strian, BCS = basal circumferential strain, GCS = global circumferential strain, EDV = end-diastolic volume, ESV = end-systolic volume, LVEF = left ventricular ejection fraction.

**Table 2:** The paired mean data across the users of the paired repetitions and software.

Variable	OsiriX: Test 1 vs Test 2					ScanIP: Test 1 vs Test 2				
	Mean of attempts	Mean Difference	SD of mean difference	SD as a % of mean	P	Mean of attempts	Mean Difference	SD of mean difference	SD as a % of mean	P
**ACS (%)**	-11.2	-1.4	5.2	46.4	0.51	-14.7	-2.5	3.2	21.8	0.08
**MCS (%)**	-19.25	-0.9	1.9	9.8	0.23	-20.85	-0.5	1.0	4.8	0.24
**BCS (%)**	-29.8	-0.6	1.6	5.4	0.38	-30.15	-0.7	2.6	8.6	0.51
**GCS (%)**	-19.85	-0.3	1.4	7.1	0.57	-20.6	-0.6	1.1	5.3	0.22
**EDV (mL)**	159.4	1.4	5.8	3.6	0.54	123.3	0.9	3.4	2.8	0.50
**ESV (mL)**	97.85	0.5	5.8	5.9	0.83	75.4	-1.2	4.2	5.6	0.47
**LVEF (%)**	38.75	0.3	1.9	4.9	0.70	38.85	1.4	2.1	5.4	0.13
	**Test 1: OsiriX vs ScanIP**					**Test 2: OsiriX vs ScanIP**				
**ACS (%)**	-13.9	4	4.5	32.4	0.06	-12	2.9	1.3	10.8	1e-3
**MCS (%)**	-20.4	1.3	2.2	10.8	0.15	-19.7	1.8	1.7	8.6	0.03
**BCS (%)**	-30.3	0.4	3.2	10.6	0.75	-29.65	0.3	2.5	8.4	0.78
**GCS (%)**	-20.45	0.9	2.0	9.7	0.30	-20	0.6	2.0	10.0	0.44
**EDV (mL)**	141.95	36.3	8.2	5.8	1e-5	140.75	35.8	13.3	9.4	4e-4
**ESV (mL)**	86.45	23.3	10.7	12.4	1.2e-3	86.8	21.6	13.8	15.9	6e-3
**LVEF (%)**	39.25	-0.7	4.6	11.7	0.69	38.35	0.4	4.4	11.5	0.80

Mean paired differences between groups, and the standard devations of the the mean paired differences, and the standard deviation as a percentage of the mean are also shown. P-values are from the two sets of paired t-tests are also presented, and are highlighted in red where there are changes significant to the Bonferroni corrected value of p<0.007.

The magnitude of the standard deviation of a variable relative to the corresponding mean for a particular software and attempt gives an indication of the reproducibility of that variable between users. For instance, the reproducibility between users for the strain variables MCS, BCS, and GCS was good, with the standard deviations (SD) for these three variables being less than 10.4% of the mean value for all groups (Table 1). The reproducibility for the volume metrics was also good, with all SDs being less than 10.6% of the mean value for the groups. However, for the strain variable ACS the reproducibility was lower, with the SD being a maximum of 34% of the mean value across the groups.

### Paired group statistics

Paired means, and mean differences between the four groups and their standard deviations, are presented in Table 2. Standard deviations of the paired differences were on average higher for the differences between the two software packages, rather than between reader test repetitions on the same software. The repeatability between OsiriX tests 1 and 2, and ScanIP tests 1 and 2 were good for all variables with all mean difference SDs being less than 10% of the mean values, apart from ACS, where the mean differences SDs were 46% for OsiriX, and 22% for ScanIP. The repeatability between a given attempt between software packages (inter-software) was lower than the intra-software repeatability, with all mean difference SDs being less than 16% of the mean values, apart from ACS, where the mean differences SDs were less than 32% of the mean values.

**Fig 3:**
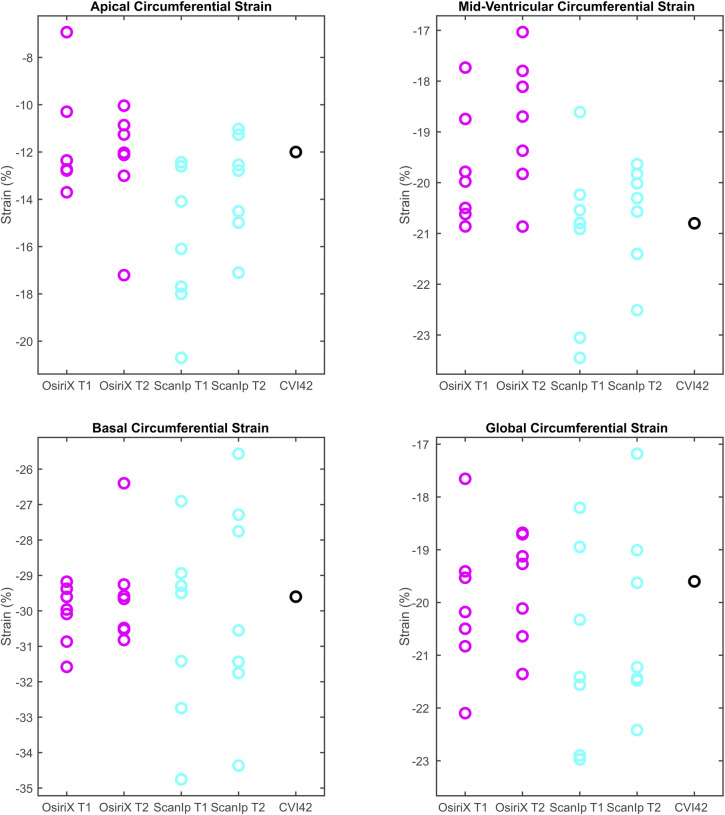
Boxplots of peak data for the four circumferential strain variables investigated. a) apical circumferential strain, b) mid-ventricular circumferential strain, c) basal circumferential strain, d) global circumferential strain. The circles represent data from individual readers. T1 and T2 are the first and second repetition of contouring in a given software. Equivalent data from CVI42 is shown in the fifth column.

**Fig 4:**
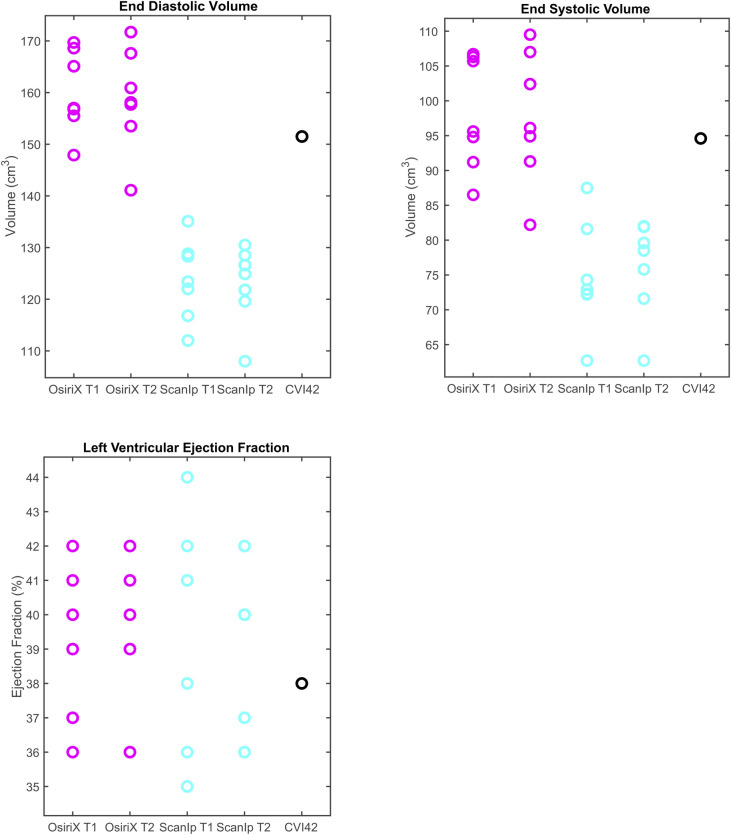
Boxplots of peak data for the three clinical volume variables investigated. a) end diastolic volume, b) end systolic volume, c) left ventricular ejection fraction. The circles represent data from individual readers. Equivalent data from CVI42 is shown in the fifth column.

The results for all variables from the CVI42-based method lay within the same ranges for the equivalent variables when calculated using OsiriX (Figs 3A–3D & 4A–4C). For the relative metrics (strains and LVEF), the CVI42 data was generally within the ranges for both ScanIP and OsiriX (Figs 3A–3D & 4C). For the absolute volume metrics, EDV and ESV, the CVI42 data was only in the ranges for the OsiriX tests (Fig 4A and 4B).

Investigation of the paired t-tests showed that EDV had highly statistically significant differences between the two software packages for both sets of repetitions (both p≤0.0004, Table 2). The ESV measurement also showed statistically significant differences between OsiriX and ScanIP for both repetitions (both p≤0.006, Table 2). For ACS, the paired t-tests indicated that there was evidence of a mean difference between software packages for repetition 2 only (Table 2).

### Bland-Altman analysis of systematic differences

**Fig 5:**
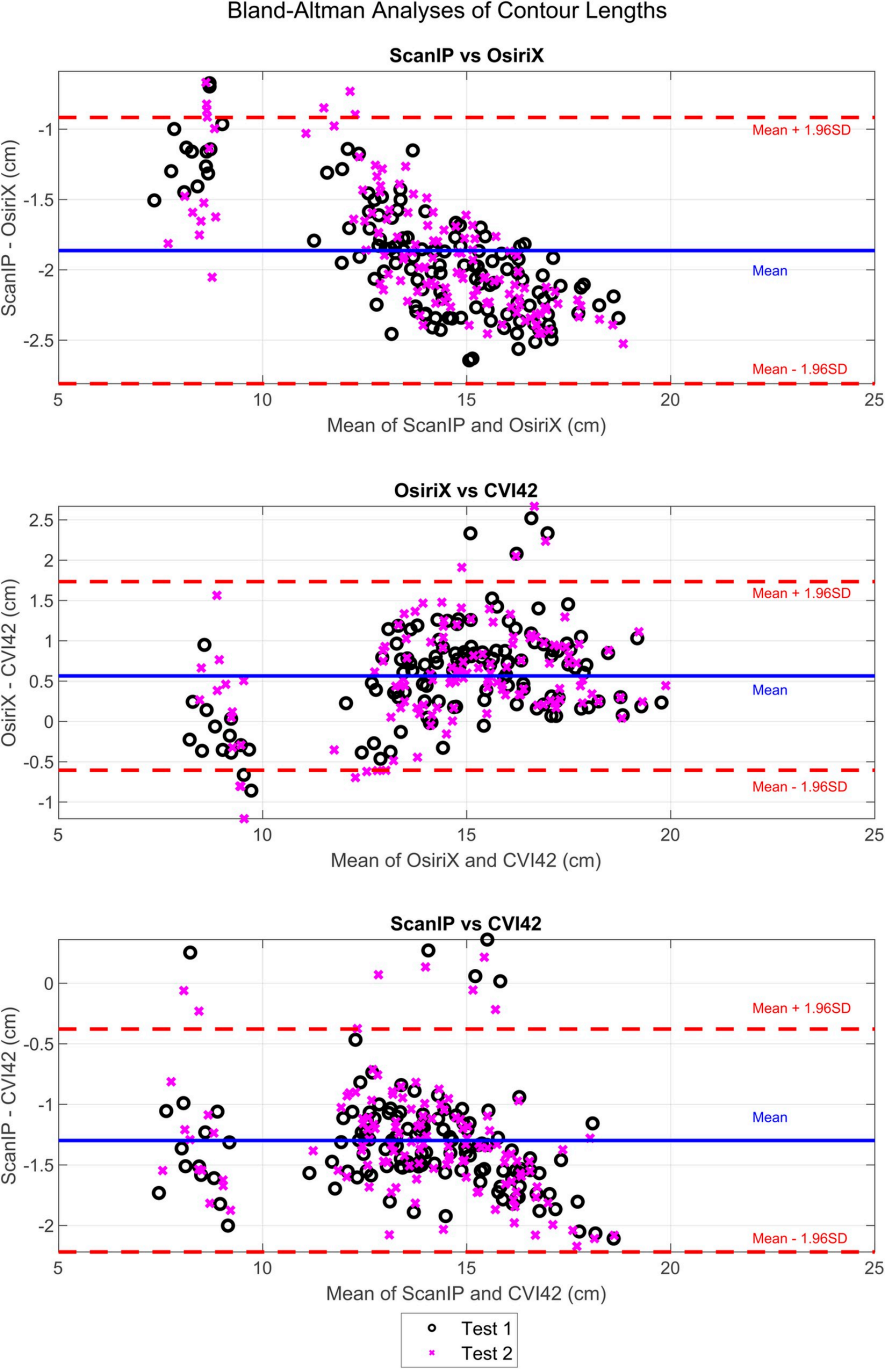
Bland-Altman analyses of differences in endocardial contour lengths measured in ScanIP, OsiriX, and CVI42. Results from test 1 and test 2 from ScanIP and OsiriX are shown. The CVI42 data was assumed to be 100% repeatable, and thus the same data was used for both tests 1 and 2, as previously described.

Bland-Altman analysis of the contour length measurements showed that there was a systematic difference in the lengths between all three pieces of software (Fig 5A–5C). For ScanIP vs OsiriX and ScanIP vs CVI42, trends were seen; as the mean increased, the magnitude of the difference increased. There was a mean bias of -1.9 cm towards ScanIP when compared to OsiriX. There was a mean bias of +0.6 cm towards OsiriX when compared to CVI42. There was a mean bias of -1.3 cm towards ScanIP when compared to CVI42. This indicates that perimeter lengths were estimated to be longer than OsiriX, when using ScanIP, and slightly shorter than OsiriX when using CVI42.

## Discussion

This study aimed to investigate variability in MRI-derived LV metrics measured using different software packages and by different readers. The main findings are firstly that all metrics apart from ACS were reproducible, and all repeatable within a given software. ESV and EDV were more affected by choice of software package rather than by intra-software variability, indicating they can be influenced by choice of software package.

Statistically significant differences in absolute metrics EDV and ESV did not result in significant differences for relative metrics (strains and LVEF). Systematic errors present in the absolute metrics were ‘cancelled out’ in the relative metrics making them repeatable across software packages. This occurred as, on average, if a user had higher EDV values they also had larger ESV values, and the same was true for smaller ESV and EDV values for a given user. When calculating LVEF ((EDV-ESV)/EDV), the stroke volume (EDV-ESV) remained approximately consistent, whether ‘over’ or ‘underestimating’ the volumes. In this manner, the relative metrics LVEF (and also the strains) were more repeatable as the systematic ‘over’ or ‘underestimation’ was cancelled out.

This effect was particularly strong for the global metrics GCS and LVEF, in which differences for individual contours cancelled out over the whole ventricle. For MCS and BCS no statistically significant differences were found at either the software or attempt level, suggesting there was no bias in the mean values. For ACS, a difference between OsiriX test two and ScanIP test two was found (p = 0.001, Table 2). Standard deviations for group means, and for group mean differences, were higher for ACS than for other strain metrics. However, no statistical differences were found in the other paired t-tests for ACS, suggesting a lack of bias.

As mentioned earlier, variability in contour placement was most pronounced at the apex in all three software packages; this variability at the apex has also been shown in another study [11]. The endocardial border at the apex may have been particularly difficult for CVI42 to capture, as the shape of the porcine LV is slightly different to that of humans [23, 24], the trabeculations are rougher [23], and the software was trained only for use on human data. Apical contouring in general could be improved with additional training of the readers, however, as shown in Suinesiaputra et al. [11] contour placement is to a certain extent subjective in nature, even between expert readers. It was noted by one user (User 7) who had not used either ScanIP or OsiriX before this study, that the ScanIP GUI was harder to use for contouring than the OsiriX GUI. However, the paired differences in clinical metrics did not show that this user performed any worse than other users on either software package.

Small mean differences between most groups for most variables (apart from ACS), together with the relatively small standard deviation of the mean differences in comparison to the paired groups means showed that intra-software repeatability was higher than inter-software repeatability (Table 2) which is consistent with other studies of this nature [10, 18]. The CVI42 method found all metrics to be in the same ranges as those found by OsiriX tests 1 and 2. The main data used to calculate these metrics were contour lengths, which were shown, by the Bland-Altman analysis, to be repeatable across these two software packages, with a small mean difference of 0.6 cm (Fig 5B). Both packages use a spline curve to represent the contour, and the OsiriX reader-averaged contour length was 0.6 cm longer than those found by the automated CVI42. Thus, the strain and volume calculation techniques yielded consistent results between the modified CVI42 and OsiriX software packages.

Clearly certain results were affected by the software package, most noticeably with Bland-Altman analysis showing there were systematic differences between contour lengths measured in ScanIP and OsiriX, with a mean bias of -1.9 cm to ScanIP (Fig 5A), and contour length differences between ScanIP and CVI42, with a mean bias of -1.3 cm to ScanIP (Fig 5C). The bias in decreased contour length could be due to how the images from ScanIP were generated and analysed. The binarised images outputted from ScanIP were pixelated and calculating the convex hull of the points likely led to an underestimation in length. The trends seen in both the ScanIP vs OsiriX plot (Fig 5A) and ScanIP vs CVI42 plot (Fig 5C), indicated that the bias between methods changed over the range of values, i.e. the variability of differences was larger when the value being measured was larger. This issue did not arise between OsiriX and CVI42, suggesting that ScanIP may have greater variability associated with its measurements. Although this could indicate the mean difference between measurements from the two software packages varies with the underlying value being measured, such a trend can also be caused simply by the measurement error variances for the two packages differing [25]. These results also suggest that contour length was not as repeatable between ScanIP and OsiriX, and ScanIP and CVI42, as between OsiriX and CVI42, due to the larger mean biases seen when making comparisons to ScanIP.

EDV and ESV are used as absolute measures, as well as measures of temporal change, to guide pharmacotherapy, device implantation, and surgical intervention [19]. Large differences in volumes could therefore have important clinical implications. Typically, errors in volume of up to 8% are expected clinically [22]. It has been shown by Miller et al. (2012), that using the same EDV and ESV contouring and slice volume summing technique as presented here, both EDV and ESV were overestimated, leading to an underestimation in LVEF. This led to 25% of their cohort being assigned to different LVEF severity categories, when compared to data resulting from contouring defined in a more detailed manner (precise delineation of trabeculae, and non-inclusion of papillary muscles in LV cavity volume). In this study, the percentage errors (based on standard deviations) in EDV were found to be a maximum of 6.3%, and for ESV a maximum of 10.6%. Larger errors are expected in ESV due to the technical challenge of tracing the compacted myocardium [19]. These results suggest that the LVEFs may be overestimated, leading to clinical decisions based purely on volumetric measures being incorrect or misclassified. In this study, relative metrics such as strains and LVEF showed better overall reproducibility and repeatability and were ultimately more robust as metrics. However, similarly to volume estimation techniques, repeatability is reduced when using different software packages to calculate strains, as inconsistencies arise due to differing algorithms [10, 19].

It is also possible that the context in which the images were viewed, contributed to differences in manual contour placement. In OsiriX, the same cine-slice was seen through 16 points in the cardiac cycle, whereas in ScanIP all slices from one time-point were seen in sequence. It has been noted elsewhere that the context of an image, either viewing one slice through time (2D), or considering 3D methods segmenting multiple slices a volume, changes the resultant perception of the contour whether contouring is done manually or automatically [26].

There are limitations to this study. Only one porcine left ventricle was investigated, however the total number of image stacks (16) and individual images (144) that were analysed is comparable to other studies [9, 11]. As these image stacks were taken throughout the cardiac cycle the variation between individual images is high, thereby providing a robust test case for the users. Finally, this particular specimen was suffering from heart failure and reproducibility in strain has been shown to be greater for human patients with heart failure rather than healthy volunteers [18, 27]. Investigation of the repeatability of this metric on other specimens and for other pathologies would therefore be valuable.

The sample of readers (n = 7) was small, however, this is comparable to the number of readers in similar studies [9, 11] and all p-values have had Bonferroni corrections applied, reducing the likelihood of false positives. Finally, CVI42 is not currently validated for use in animals; however, the automatically generated contours were all visually checked and in practice found to be clinically acceptable and only required correction at the apex, which is known to be a difficult region to contour in humans [11].

## Conclusion

This study investigated the repeatability of ventricular circumferential contours, and their derived volume and strain metrics, for a porcine heart failure model. Two software packages were used by seven readers and on two separate occasions, and a further automated software was also tested on one occasion. No statistically significant difference in circumferential strain measurements (BSC, MCS and GCS), or relative volume (LVEF), were found (all p>0.007), despite systematic biases in contour length between the software packages OsiriX and ScanIP. However, these biases did result in differences in EDV and ESV values between software packages. Differences between software packages were also found for ACS due to the difficulties in capturing the shape of the apex. Intra-software differences for variables were found to be lower than inter-software differences. These results will benefit future studies that use contouring methods and volume & strain metrics derived from these contours, by providing associated error estimates, guidance to improve repeatability, and aiding comparison of results with other studies.
